# Mechanistic Role of TRIM26 in Viral Infection and Host Defense

**DOI:** 10.3390/genes15111476

**Published:** 2024-11-15

**Authors:** Mona Sharma, Ke Liu, Jianchao Wei, Zhiyong Ma, Yafeng Qiu

**Affiliations:** Shanghai Veterinary Research Institute, Chinese Academy of Agricultural Sciences, 518 Ziyue Road, Shanghai 200241, China; monasharm1990@yahoo.com (M.S.); liuke@shvri.ac.cn (K.L.); jianchaowei@shvri.ac.cn (J.W.); zhiyongma@shvri.ac.cn (Z.M.)

**Keywords:** Tripartite motif protein 26 (TRIM26), viruses, antiviral response

## Abstract

*Tripartite motif protein 26* (TRIM26) is an E3 ubiquitin ligase and a member of the TRIM family. Similar to other TRIM proteins, TRIM26 consists of three domains, collectively termed RBCC: a Really Interesting New Gene (RING) domain, one B-Box domain, and a C terminal domain consisting of a PRY/SPRY domain. The PRY/SPRY domain exhibits relatively higher conservation compared with the RING and B-Box domains, suggesting potentially similar roles across TRIM26 proteins from various species. TRIM26 either directly interacts with viral proteins or modulates immune responses to engage with a viral infection, serving as either a protective or detrimental host factor depending on the circumvent of the viral infection. The present review focuses on understanding the mechanisms of TRIM26 during viral infection and its potential future applications.

## 1. Introduction

TRIM proteins are a subfamily within the RING-type E3 ubiquitin ligase family, which plays a key role in various cellular processes, including innate immune responses. They are involved in cell signaling pathways that trigger cytokine production and antiviral defenses [1]. Numerous TRIM molecules were recently shown to mediate antiviral innate immune responses [2], but the underlying molecular mechanisms remain elusive. A protein that is a member of the tripartite motif (TRIM) family, encoded by the TRIM26 gene, has three zinc-binding domains: the RING domain, one B-Box domain, and a coiled-coil CC domain followed by a C terminal domain consisting of a PRY/SPRY domain. This protein is typically found in both cytoplasmic and nuclear bodies. While its precise function remains unclear or controversial, the presence of a RING domain suggests potential binding activity to target proteins, leading to proteasomal degradation. The gene is located on chromosome 6 in the major histocompatibility complex (MHC) class I region [2,3]. Different transcript variants with alternative splicing that encode the same protein have been reported. In humans, the TRIM26 gene is found on chromosome 6p22 [2], on chromosome 17 in house mice [1], and on chromosome 7 in *Sus scrofa* (pig) [4]. Additionally, TRIM26 has been involved in various cellular processes, including tumorigenesis in different cancer types, autophagy, inflammatory response, and the innate immune signaling pathway [1]. Also, TRIM26 has been reported for its versatile role as both protective and detrimental depending on the circumvents of various viral infections. Therefore, exploring the structure of TRIM26 in comparison with other TRIM molecules, including TRIM26 in other species, is of major importance. In this review article, we provide a detailed analysis of TRIM26’s structure and function, discussing known cellular processes and molecular mechanisms conferring the antiviral response, current constraints, and potential future applications, focusing primarily on TRIM26’s role in the antiviral response across various cellular contexts.

## 2. Structural and Sequence Characteristics of TRIM26

**Figure 1:**
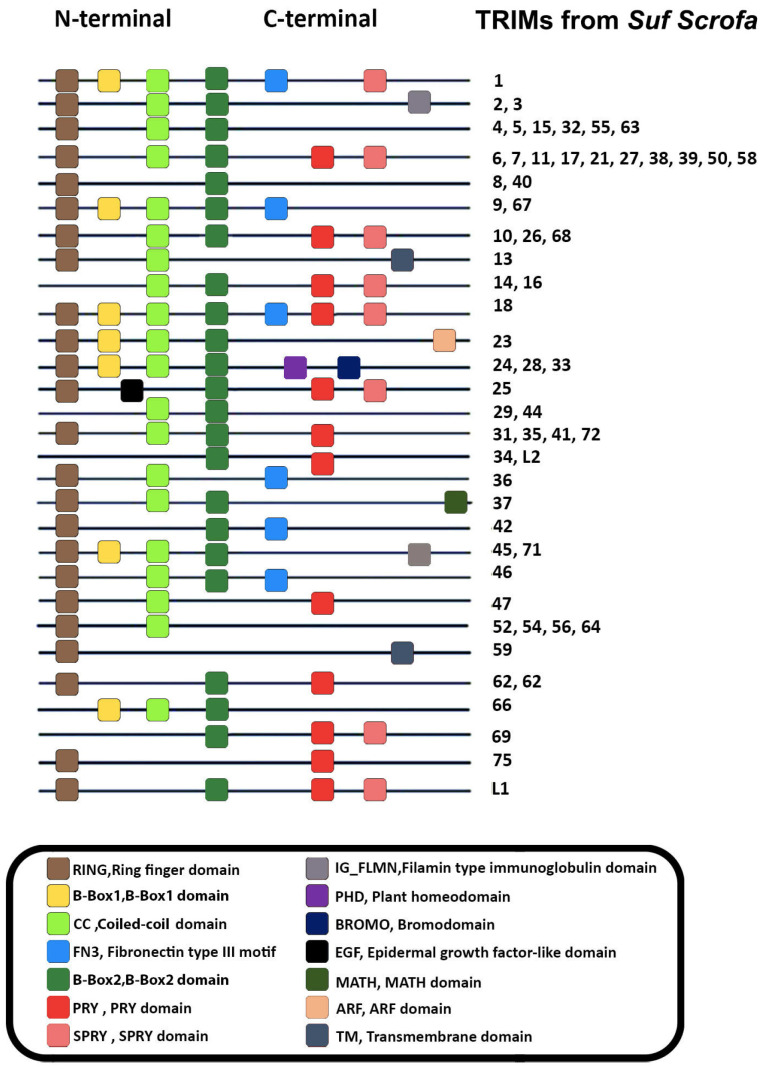
TRIMs derived from *Suf Scrofa* (pigs) structures. TRIM sequences from pigs were examined and potential domains were inferred. TRIMs from pigs have a conservative N-terminal. The C-terminal, on the other hand, has a range of variable domains.

TRIMs are E3 ubiquitin ligase family proteins, which are present in almost all mammals [1,2]. The amino-terminal region of the TRIM protein contains three domains that are considered relatively conservative (RBCC): the coiled-coil domain (CC), the B-Box domain (BBox), and the Really Interesting New Gene (RING) domain. Cysteine, histidine, and zinc comprise the RING domain, a zinc finger motif that gives TRIMs their E3 ubiquitin ligase activity. Cysteine histidine–zinc binding motifs are also present in the B-Box domains, which include B-Box1 and/or B-Box2. Several α helices make up the supercoiled CC domain of TRIM, which is essential for the formation of TRIM homologous polymers. Likewise, research indicates that the N-terminal of porcine TRIMs is conservative based on TRIM sequences in animals [2,4]. The RING domain of the protein raises the possibility that it has DNA-binding ability, even though its exact function is not very well known [5]. While TRIMs are highly conserved across species, they might consist of different domains in the same species. For instance, we identified that the N-terminal of porcine TRIMs is conservative based on TRIM sequences in animals (Figure 1). On the other hand, there are numerous variable domains at the C-terminal. It is noteworthy to mention that the majority of swine TRIMs feature RBCC-PRY/SPRY domains; motifs identical to these may be found in porcine TRIMs 10, 26, and 68. These TRIMs have one PRY/SPRY domain in the C-terminal region, one B-Box motif in the N-terminal, and one RING motif in the N-terminal.

**Figure 2:**
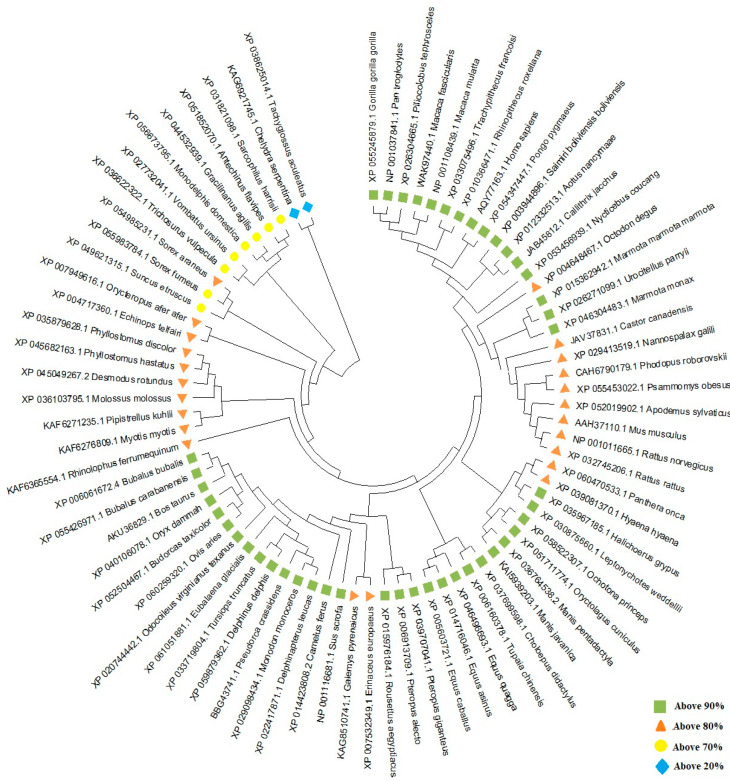
Phylogenetic analysis of TRIM26 depicting sequence divergences between different species. The amino acid sequences of 78 different species and porcine TRIM26 were compared using MEGA 11. Homologies above 90%, 80%, 70%, and 20% are shown above.

**Figure 3:**
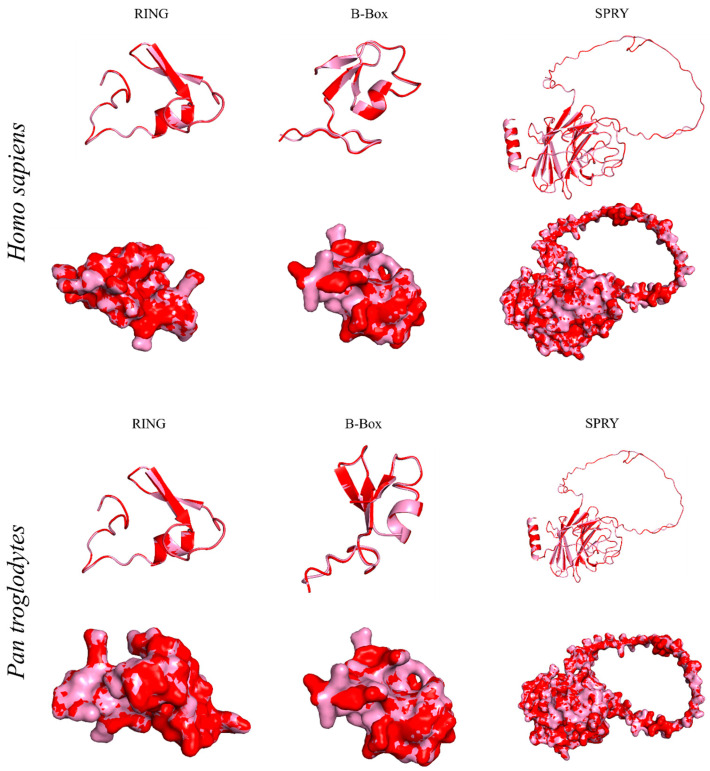
Predicted structural similarity between porcine TRIM26 and TRIM26 of *Homo sapiens* and *Pan troglodytes*. Porcine TRIM26 is shown by the color red, while *Homo sapiens* and *Pan troglodytes* TRIM26 are indicated by the color pale pink.

**Figure 4:**
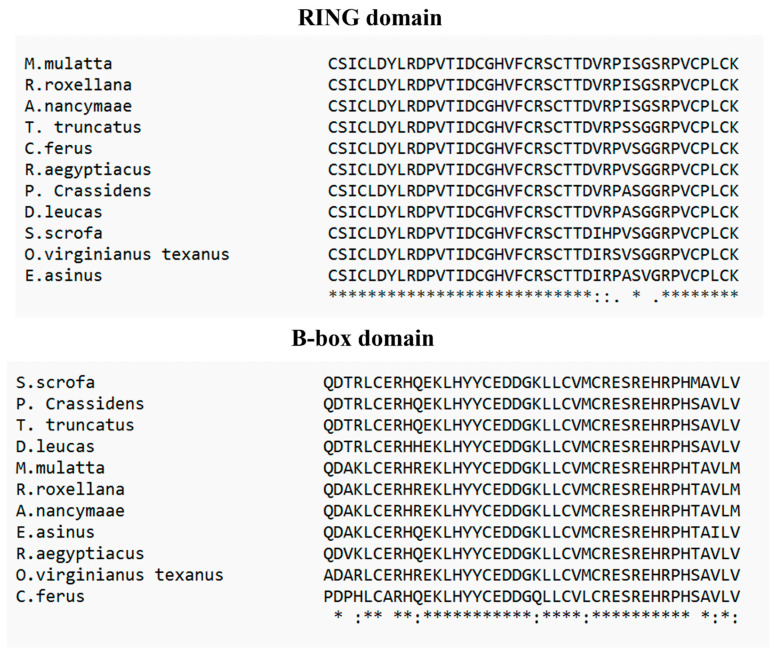
RING domain and B-Box domain comparsion of TRIM26 of *Sus scrofa* with 10 other species. ‘*’ shows a highly conserved residue, ‘:’ shows a residue that shares some similar biochemical properties, and ‘.’ shows little similarity between the aligned sequences.

TRIM26, also known as AFP, RNF95, or ZNF173, underwent a comprehensive comparative analysis with 78 different species using Molecular Evolutionary Genetic Analysis (MEGA) version 11 (Figure 2) [6]. The results reveal a significant homology of over 90% between porcine TRIM26 and 45 other species, and a homology exceeding 80% with 23 additional species. Particularly, amino acid sequences of TRIM26 from *Eubalaena glacialis* (XP_061051881.1), *Delphinus delphis* (XP_059879362.1), and *Pseudorca crassidens* (BBG43741.1) were found to share high degrees of homology, exhibiting percentages of 93.33%, 93.14%, and 93.14%, respectively. Interestingly, porcine TRIM26 demonstrates a homology with its human and murine counterparts, with 92.39% and 84.40% similarities, respectively. A detailed breakdown of TRIM26 homology with 44 other species, each showing homology above 90%, is provided in Table 1, offering a comprehensive overview of the evolutionary relationships of TRIM26 across a diverse set of organisms. Furthermore, we dissected the TRIM26 using PyMOL, a popular tool for the three-dimensional (3D) visualization of proteins, nucleic acids, small molecules, electron densities, surfaces, and trajectories [7]. Porcine TRIM26 and TRIM26 of the uniport-reviewed species, *Homo sapiens*, have comparable domains. For comparison. *H.sapiens* (Id: Q12899) and *Pan troglodytes* (Id: Q7YR34) were selected. The domain sequences of *H.sapiens*, *P. troglodytes*, and *Sus scrofa* were analyzed. Due to the lack of structural information, homology modeling was employed to predict the three-dimensional structures of the respective domains. The comparison of two TRIM proteins is visually depicted in Figure 3, Figure 4 andFigure 5. The structural similarities between the domains of *H.sapiens* and *P. troglodytes* are contrasted with that of *S.scrofa* (Figure 3). The predicted structure, generated by the Alpha Fold model, emphasizes a notably high similarity in TRIM26 between these two species. To conduct a comprehensive analysis, multiple sequence alignment was performed using ClustalW [8] by aligning domain sequences from ten distinct species. Figure 3 and Figure 4 provide a graphical representation of the conserved regions within TRIM26 across various species. This dual approach, combining structural insights from Figure 3 and sequence conservation from Figure 4 andFigure 5, contributes to a more thorough understanding of the similarities and conservation patterns exhibited by TRIM26 protein across a diverse set of organisms. These findings suggested a greater degree of conservation of TRIM26 across numerous species. The evolutionary stability observed in these domains among the phylogenetically diverse organisms not only adds substantial support to the notion of TRIM26 functional similarity or the equivalent in various species but also underscores its conserved nature. This broad conservation of TRIM26 domains across species further indicates a functional similarity or even identity in multiple organisms. In essence, the findings highlight the consistent and potentially universal role of TRIM26 in various biological contexts across different species.

## 3. Functional Characteristics of TRIM26

**Figure 6:**
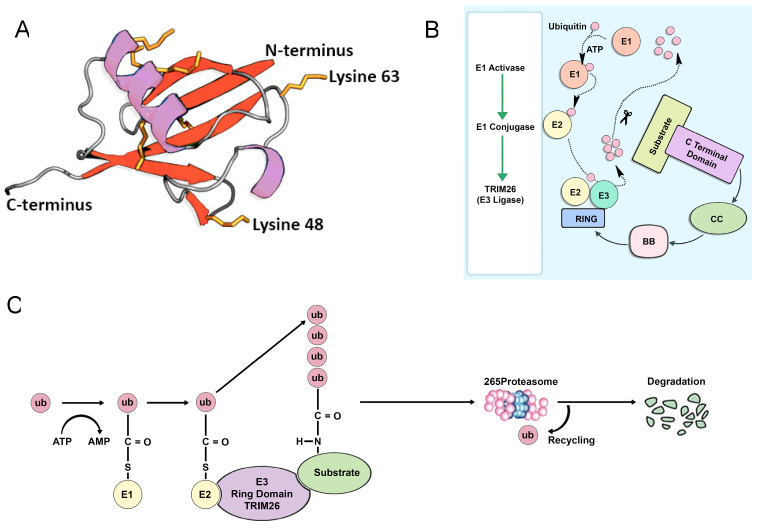
Overview of TRIM26 function. (**A**) E3 ligase ubiquitin, a tiny protein with 76 amino acids that is highly conserved in eukaryotic species, is a part of the structure of TRIM26. One of the seven lysine (K) residues on target proteins, such as 48 or 63, can be linked to E1 ubiquitin, which functions as a flag for endocytosis, DNA repair, and enzymatic activation, in addition to proteasome-mediated destruction. (**B**) The conjugation reaction of ubiquitin is catalyzed by the E1 ubiquitin-activating enzyme, E2 ubiquitin-conjugating enzymes, and E3 ubiquitin ligases. (**C**) E3 functions as a scaffold that mediates between E2 and the substrate, which is in charge of substrate recognition. This is the biochemical reaction of the ubiquitin proteasome activity. The 26S proteasome then finds the resultant polyubiquitinated conjugates and quickly breaks them down.

TRIM26 proteins are pivotal in regulating essential cellular processes, such as innate immunity, intracellular signaling, transcription, carcinogenesis, and autophagy [9,10]. TRIM proteins are primarily recognized for their involvement in proteolytic degradation via ubiquitination [10]. Ubiquitination is a cascade of reactions catalyzed initially by E1 ubiquitin-activating enzymes; followed by E2 ubiquitin-conjugating enzymes; and finally, the crucial E3 ubiquitin ligases, which directly recognize substrates. Over eighty genes encoding TRIMs have been identified in humans [9]. While most TRIM family members, including TRIM26, are classified as E3 ubiquitin ligases due to the presence of a RING finger domain, it is notable that not all TRIM proteins possess this domain; eight TRIM proteins in humans lack a RING finger domain. E3 ubiquitin ligases, consisting of approximately 76 amino acid residues, have a carboxy terminus that is conjugated to the ε-amino group of a lysine (K) residue of the target protein via an isopeptide bond, as illustrated for TRIM26 in Figure 6A. This conjugation leads to substrate recognition and subsequent degradation, underscoring the importance of TRIMs, such as TRIM26, in intracellular signaling pathways and processes. To delve deeper into the function of TRIMs, it is crucial to understand that E1 ubiquitin-activating enzymes, E2 ubiquitin-conjugating enzymes, and E3 ubiquitin ligases catalyze the activation conjugation reaction of ubiquitin, which constitutes the functional activity of TRIM26 [11]. This reaction is initiated when the TRIM26 E3 ligase at the protein site detects a substrate. To enable E3 ubiquitin ligase to recognize substrates and transfer ubiquitin from E2 ubiquitin-conjugating enzymes to substrates, the polyubiquitinated substrates are subsequently degraded by proteasomes, as depicted in Figure 6B,C.

## 4. Role of TRIM26 in Viral Infection

In addition to their unique mRNA expression patterns during viral infection, various members of the TRIMs play a crucial role in modulating the antiviral response, acting either as suppressors or enhancers in different viral infection [3]. TRIM26 overexpression affects the transcription of pro-inflammatory cytokines, IFN-stimulated genes (ISGs), and type I IFN [12]. TRIM26 has a role in viral infection by inherently limiting the spread of viral infections and enhancing immunological pathways to aid in the elimination of viruses [13]. However, a report suggests that TRIM26 also plays a role in suppressing antiviral pathways to help the virus to evade the immune response, leading to persistent infection [3]. Therefore, the role of TRIM26 proteins varies, either being protective or detrimental in different viral infection scenarios, highlighting the importance of dissecting TRIM26’s role in viral infection for potential therapeutic use as an antiviral strategy. Furthermore, TRIM26 employs numerous mechanisms through which it can either negatively or positively affect various viral infections, as described below.

### 4.1. Hepatitis C Virus (HCV) Infection

Liang et al. (2021) identified TRIM26 as an essential host factor for HCV replication, showing that the absence of TRIM26 significantly impairs the virus’s ability to replicate its genome [14]. Their research demonstrated that TRIM26 promotes K27-linked ubiquitination on lysine 51 (K51) of HCV’s NS5B protein, a process that strengthens the interaction between viral proteins NS5B and NS5A. This enhanced interaction plays a critical role in advancing HCV replication while modulating the immune response to the virus [14]. Further experiments revealed that overexpressing TRIM26 in a mouse hepatoma cell line, along with other host factors essential for HCV, increased the efficiency of HCV infection, highlighting TRIM26’s contribution to host tropism. By facilitating viral protein interactions and supporting key stages in the HCV replication cycle, TRIM26 not only aids viral replication but also influences how the host immune system responds to the virus. These findings underscore the importance of TRIM26 in HCV’s life cycle and its potential as a therapeutic target. Therefore, exploring this study could be very interesting for identifying novel therapies.

### 4.2. Herpes Simplex Type 2 Virus (HSV-2) Infection

**Figure 7:**
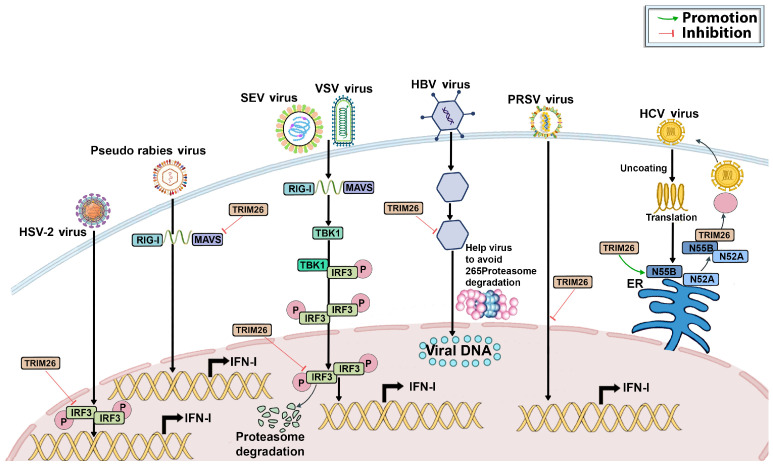
TRIM26 functions as a suppressor of immune responses during a viral infection. The ubiquitination of IRF3 or its upstream sensors by TRIM26 results in immune suppression during viral infection. Ultimately, the replication of viruses, such as HSV-2, PRV, SEV, VSV, HBV, HCV, and PRRSV, is facilitated in a TRIM26-dependent manner.

Genital herpes is primarily caused by HSV-2, a virus responsible for significant morbidity and mortality worldwide, especially among women. HSV-2 primarily spreads through infection of epithelial cells on the skin and mucosal surfaces [15,16]. In a study by Dhawan et al. (2021), the role of TRIM26 in HSV-2 infection was investigated using human vaginal epithelial cells (VK2). Their research revealed that HSV-2 infection significantly increases TRIM26 production in these cells due to interactions between the virus and vaginal epithelial cells [17,18]. Further experiments on TRIM26 expression showed that overexpression of TRIM26 in VK2 cells led to a substantial increase in viral replication, whereas TRIM26 deficiency resulted in limited HSV-2 replication. In addition, researchers measured interferon-β levels and the expression of two interferon-stimulated genes (ISGs), MX1 and ISG15, in cells both before and after HSV-2 infection. They found that TRIM26 deficiency led to a marked increase in interferon-β production both at baseline and following HSV-2 infection. Immunofluorescence studies also demonstrated that high levels of TRIM26 significantly reduced the nuclear presence of IRF3, the primary activator of ISGs, both before and after HSV-2 infection [4,18]. These findings suggest that HSV-2 leverages TRIM26 as a host factor to enhance its replication in vaginal epithelial cells by dampening the host’s antiviral immune response (Figure 7). TRIM26 may represent a promising target for mitigating HSV-2 infection, particularly in women. Further investigation into TRIM26 could enhance our understanding of the signaling pathways that lead to viral replication.

### 4.3. Pseudorabies Virus (PRV) Infection

PRV has developed many tactics to avoid the antiviral defense system of the host, which helps the virus reproduce and build a long-lasting infection. TRIM26 is known to play an important role in innate immunity, particularly in modulating viral infections [19,20]. Studies have observed a significant upregulation of TRIM26 expression following PRV infection [19]. Interestingly, increased expression of TRIM26 appears to promote PRV replication, whereas reducing TRIM26 levels impedes viral replication, suggesting a positive regulatory role of TRIM26 in PRV infection. Further investigation revealed that TRIM26 negatively impacts the innate immune response by specifically targeting the type I interferon-signaling pathway, which is activated by RIG-I. TRIM26 was found to interact with MAVS (mitochondrial antiviral-signaling protein) regardless of viral presence, resulting in a downregulation of MAVS expression. Mechanistically, it was shown that NDP52 interacts with both TRIM26 and MAVS [19]. When NDP52 was knocked down in cells, the degradation of MAVS by TRIM26 was significantly reduced. These findings suggest that TRIM26 mediates MAVS degradation through NDP52-dependent selective autophagy, thereby facilitating viral immune evasion.

### 4.4. Sendai Virus (SEV) and Vesicular Stomatitis Virus (VSV) Infection

**Figure 8:**
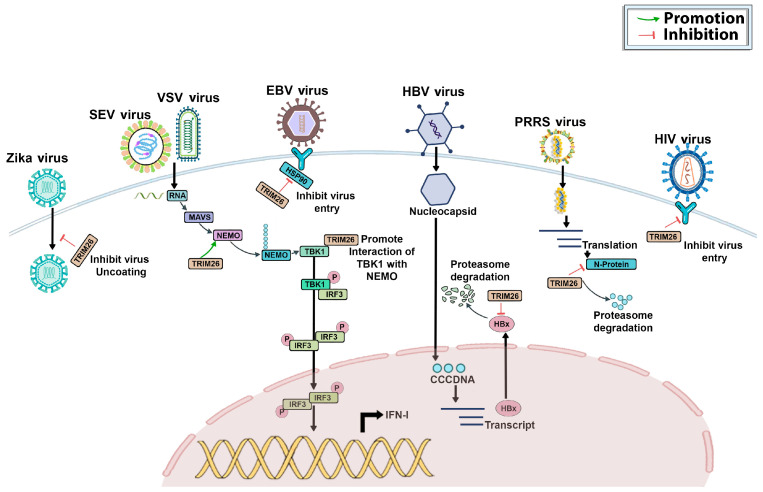
TRIM26 exerts a beneficial impact on the host immune responses during a viral infection. TRIM26 displays versatile roles across different viral infections, modulating the regulation of type I IFN during diverse viral challenges.

In general, the presence of a virus in the body activates a transcription factor called IRF3, leading to the production of type I interferons. These interferons stimulate the transcription of ISGs, which work to eliminate the viral infection [21,22]. The activation process of IRF3 involves dimer formation, phosphorylation, and nuclear translocation. However, the mechanisms by which the nuclear activation of IRF3 is terminated remain unclear [23]. It has been reported that TRIM26 hinders the synthesis of IFN-β and the antiviral response by specifically targeting nuclear IRF3 in the presence of an RNA viral infection, like SeV or VSV, as shown in Figure 7. TRIM26 enhances the accumulation of K48-linked polyubiquitination in the nucleus and facilitates the degradation of IRF3 [24]. Notably, TRIM26 does not degrade the phosphorylation-deficient mutant 5A, but it does enhance the degradation of the constitutively active mutant IRF3 5D and wild-type (WT) IRF3. Consequently, TRIM26 inhibits IFN-β production and promoter activation following the TLR3/4-, RLR-, and DNA-sensing pathways, resulting in increased viral replication and decreased IRF3 activation and IFN-β production [25]. Thus, TRIM26-mediated processes specifically promote IRF3 degradation and ubiquitination in the nucleus, terminating IRF3 activation [3]. Collectively, these findings indicate that TRIM26 negatively modulates the type I interferon response by degrading molecules essential for type I interferon production. Further studies have reported that TBK1 is a crucial kinase facilitating IRF3 phosphorylation and activation after the engagement of various pattern-recognition receptors (PRRs). However, the specific processes triggering TBK1 activation remain unidentified [26]. TRIM26 controls the innate immune response triggered by RNA viruses, like SeV or VSV [26]. The suppression of TRIM26 results in enhanced IRF3 and NF-κB-activation-increased IFN-β production, and a heightened cellular antiviral response [26,27]. TRIM26 and TBK1 are physically associated, regardless of viral infection [25]. Acting as an E3 ligase, TRIM26 undergoes autoubiquitination in response to viral entry. The engagement of TBK1 to the VISA signalosome and its activation depend on ubiquitinated TRIM26 that acts as a bridge between NEMO and TBK1, facilitating their interaction, as illustrated in Figure 8. Studies reveal that TRIM26 plays a crucial role in controlling the body’s natural defense mechanisms against SeV or VSV infections by facilitating TBK1 and NEMO interaction, leading to TBK1 activation [3,27]. Therefore, TRIM26’s interaction with other molecules may yield different outcomes, either inhibiting or promoting the virus’s life cycle, depending on the virus.

### 4.5. Porcine Reproductive and Respiratory Syndrome Virus (PRRSV) Infection

Several studies indicated that TRIM26 suppresses the generation of IFN-β and the innate antiviral response. Previous research using RNA-sequencing (RNA-seq) analysis demonstrated that PRRSV infection promotes the expression of TRIM26 in porcine cells [12]. Huang et al. (2020) reported that PRRSV infection induces TRIM26 expression in porcine alveolar macrophages (PAM), consistent with earlier RNA-seq data. ELISA data show that reducing TRIM26 expression results in increased IFN-β expression compared with PRRSV-infected negative control cells [23]. Additionally, a significant decrease in the viral titer was observed in cells transfected with TRIM26-siRNA and infected with PRRSV, compared with PRRSV-infected negative control cells [23]. This indicates that PRRSV suppresses IFN-β production and the antiviral response by inducing TRIM26 expression (Figure 7). These results confirm that TRIM26 functions as an inhibitory factor in IFN-β production and antiviral response, although the underlying mechanism remains unknown [23]. Interestingly, another study by Zhao et al. (2022) demonstrated that TRIM26 interacts with the N protein of PRRSV through its C-terminal PRY/SPRY domain. The overexpression of TRIM26 hindered PRRSV replication and triggered the degradation of the N protein (Figure 8). This antiviral action was unaffected by the presence or absence of the nuclear localization signal (NLS). However, deleting either the RING domain or the PRY/SPRY region abolished the antiviral function [24]. Using siRNA to reduce the TRIM26 levels led to increased viral RNA synthesis and the overall viral load in PAMs following PRRSV infection. Thus, to know the exact mechanisms of TRIM26 affecting PRRSV infections, further investigations are needed.

### 4.6. Hepatitis B Virus (HBV) Infection

It was observed that reducing TRIM26 levels through RNA interference (RNAi) leads to a slight reduction in HBV replication in human hepatocytes [25]. The endogenous TRIM26 protein physically interacts with the HBV core protein (HBc) via its SPRY domain, with no interaction detected with the polymerase and HBx proteins. Intriguingly, despite its role as an E3 ligase, TRIM26 was found to inhibit HBc ubiquitination. In Huh-7 cells, knocking down TRIM26 resulted in HBc degradation, which was reversed by treatment with a proteasome inhibitor. Moreover, a mutant form of TRIM26 lacking the RING domain (TRIM26ΔR), acting as a dominant negative, sequestered TRIM26 away from HBc, leading to enhanced HBc degradation. Conversely, another study by Luo et al. (2022) reported that decreased TRIM26 expression increased HBV replication and vice versa. They observed that the SPRY domain of TRIM26 interacted with HBx and degraded it (Figure 8), which in turn suppressed HBV replication [25]. It was also noted that IFN increases TRIM26 expression. The TRIM26 rs116806878 variant was found to be associated with the response to pegylated interferon- alpha (Peg IFNα) treatment in two cohorts of patients with chronic hepatitis B (CHB) [25,26]. TRIM26 suppresses HBx activity by enhancing ubiquitin-mediated degradation. Moreover, the presence of TRIM26 rs116806878 could potentially serve as an indicator for predicting the responsiveness of CHB patients to Peg IFNα treatment [26]. There is controversy regarding TRIM26’s role in HBV infection. One study (Yuki Nakaya et al., 2023) suggested that TRIM26 negatively regulates HBV infection by protecting HBc from proteasomal degradation. Conversely, other studies [25] showed that TRIM26 acts as a positive regulator of the antiviral response by enhancing ubiquitin-mediated degradation. Overall, these studies underscore how HBV exploits TRIM26. However, the relationship between TRIM26 and HBc is currently unclear, and investigating this interaction may enhance our understanding of immune modulation in the fight against HBV infection [25,26].

### 4.7. Epstein–Barr Virus (EBV) Infection

TRIM26 is encoded by a major histocompatibility complex (MHC) gene and controls the invasion of EBV into nasopharyngeal epithelial cells. A previous study demonstrated that the EBV infection enhances the expression of TRIM26 and indirectly affects EphA2, an important receptor on epithelial cells that allows EBV to enter [27]. TRIM26 was found to interact with HSP-90β, facilitating its polyubiquitination and subsequent degradation through the proteasome pathway [27]. The integrity of EphA2 was impacted and the infection of EBV was hindered as shown in Figure 8. These studies suggest that TRIM26 functions as a protective regulator of the innate immune response and could be further explored to better understand its role in antiviral responses [27].

### 4.8. Zika Virus (ZIKV) Infection

ZIKV is a medically significant virus spread by mosquitoes [28,29,30]. Due to their presence in host cell nuclei and the ability to alter gene expression, ZIKV capsid proteins are important. RNA sequencing showed that capsid proteins from six flaviviruses reduced IFN and ISG expressions [29,30]. The ZIKV capsid protein prevents TRIMs, host proteins needed to generate IFN, from attaching ubiquitin molecules to the RIG-I CARD domains. This interaction was observed in vitro and interactome investigations [29,30,31]. Some TRIM proteins directly limit viral life cycle phases, while others control antiviral cytokine responses via innate immunological sensor-induced signal transduction pathways. Scaturro et al., 2018 identified TRIM26 as a cytoplasmic protein that interacts with the capsid of the Zika virus through an integrated proteomics approach [29,30,31,32,33,34]. However, it has been reported that ISG15, TRIM5, TRIM21, TRIM22, TRIM26, and TRIM56 genes become upregulated and are implicated in preventing the ZIKV from uncoating and disassembling in human myotubes [34]. The biological importance of the interaction between the capsid and TRIM26 during ZIKV infection is still unknown [34].

### 4.9. Human Immunodeficiency Virus (HIV) Infection

Certain proteins are expressed by host cells to obstruct retroviral replication, these proteins are known as restriction factors, and they are thought to be a component of the intrinsic or innate immune system [35]. Retroviruses contain the interferon-inducible cytidine deaminase APOBEC3G, which acts as an antiviral agent during reverse transcription [36]. TRIMs are the restriction factors that inhibit reverse transcription before viral replication [37]. Multiple studies indicate that TRIM26 triggers premature uncoating of the virion by binding to the viral lattice of the capsid, which leads to its degradation through the proteasome. This process also disrupts virion budding and release. However, the precise mechanism behind these effects remains uncertain [38]. Through its interaction with the viral capsid protein, TRIM26 has been linked to the prevention of HIV-1 entry and particle release. The E3 ubiquitin ligase activity of TRIM26 suppresses HIV-1 by a mechanism that causes the viral capsid protein to become ubiquitinated and eventually degrade. By preventing the viral capsid core from properly forming, this degradation hinders the virus’s ability to enter the host cell and release its particles [37,38]. Moreover, the biological significance of the interaction between the viral capsid and TRIM26 during HIV infection remains unknown [37,38,39].

## 5. Future Prospects and Conclusions

This review highlights the critical role of TRIM26 in viral infections, where it regulates viral replication and modulates immune responses, though its effects vary with different viruses. Although studies of the nine kinds of viral infections have gained insights into how TRIM26 affects viral infections, the precise mechanisms remain to be determined. Among those published data, some findings are contradictory. For example, previous research showed that TRIM26 negatively regulates the expression of IFN-I during Sendai or vesicular stomatitis viral infections [21]. In contrast, another study reported that TRIM26 positively facilitates the interaction between TBK1 and NEMO to induce the expression of IFN-I in response to cytoplasmic dsRNA viruses [22]. Furthermore, studies showed the different effects of TRIM26 on PRRSV infection: one study reported that the modulation of IFN-I expression promotes PRRSV infection; in contrast, another study found that TRIM26 restrained PRRSV infection by the degradation of N protein. According to the current understanding, the controversial findings of TRIM26 might be attributed to the different experimental conditions, especially the different in vitro models. These discrepancies could have also partly been due to challenges in elucidating the functions of its various domains involved in interactions with target proteins. Thus, to define the functions of TRIM26, more investigations are worthy of being undertaken, particularly in vivo research, which could help elucidate this discrepancy. Additionally, it is important to explore whether the physiological roles of TRIM26 observed in vitro are consistently replicated in animal models during the natural course of infection. Structural studies and gene-editing approaches, such as creating TRIM26-deficient mouse models, could be used to modulate TRIM26 expression. This would allow for an in-depth investigation of the interactions and mechanisms between TRIM26 and viruses throughout the natural course of infection. 

In conclusion, while recent progress has been made in understanding the role of TRIM26 in virus pathogenesis, some questions still remain unresolved. Further research is needed to fully explore the potential of TRIM26 as a therapeutic target for viral infectious diseases.

## Figures and Tables

**Figure 5:**
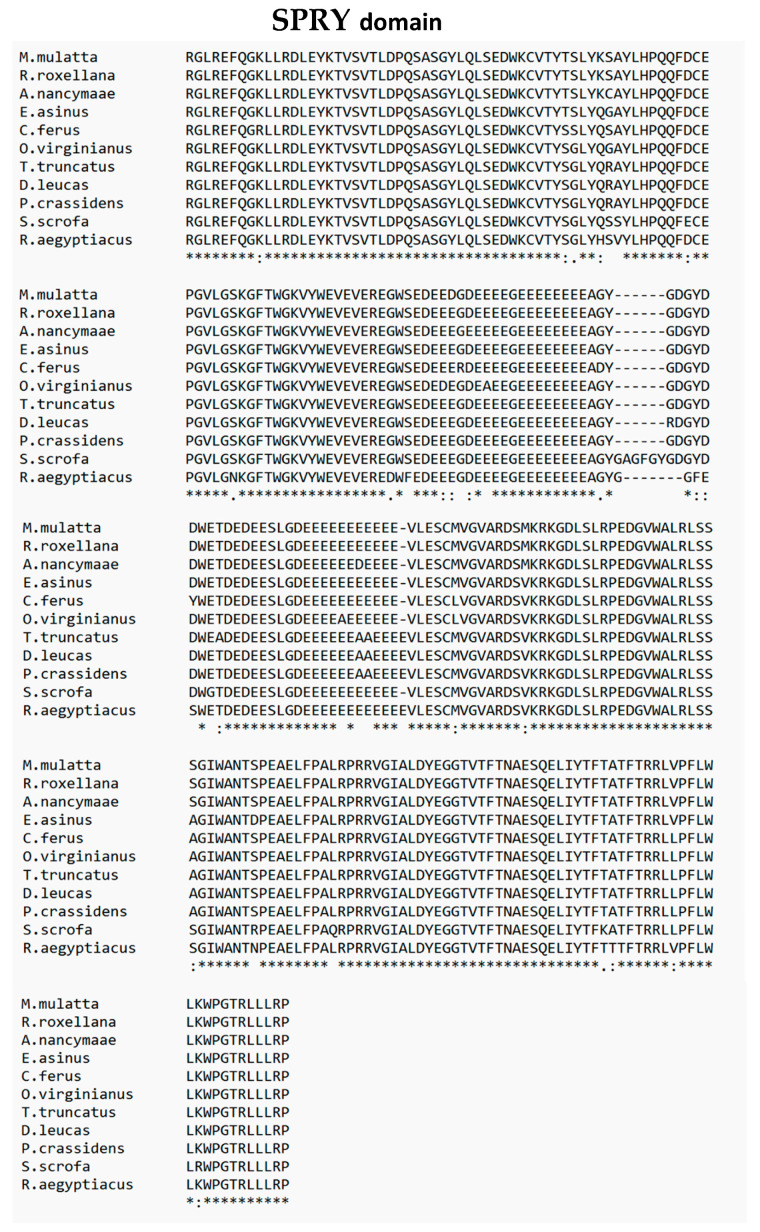
SPRY domain comparison of TRIM26 of *Sus scrofa* with 10 other species. ‘*’ shows a highly conserved residue, ‘:’ shows a residue that shares some similar biochemical properties, and ‘.’ shows little similarity between the aligned sequences.

**Table 1:** The similarity of TRIM26 to other species that have more than 90% homology.

ID	Species	Homology
NP_001116681.1	*S. scrofa* (Pig or wild boar)	100
XP_061051881.1	*E. glacialis* (North Atlantic right whale)	93.3333
XP_059879362.1	*D.delphis* (Common dolphin)	93.1481
BBG43741.1	*P. crassidens* (False killer whale)	93.1481
XP_033719804.1	*Tursiops truncatus* (Bottlenose dolphin)	92.963
XP_014423808.2	*Camelus ferus* (Wild Bactrian camel)	92.9499
XP_022417871.1	*Delphinapterus leucas* (Beluga whale)	92.7778
XP_029098434.1	*Monodon monoceros* (Narwhal)	92.7778
XP_046496693.1	*Equus quagga* (Plains zebra)	92.7644
XP_014716046.1	*Equus asinus* (Donkey)	92.7644
XP_005603721.1	*Equus caballus* (Horse)	92.7644
XP_051711774.1	*Oryctolagus cuniculus* (Rabbit)	92.5788
XP_006061672.4	*Bubalus bubalis* (Water buffalo)	92.5788
XP_020744442.1	*Odocoileus virginianus texanus* (Texas white-tailed deer)	92.5788
AQY77163.1	*H.sapiens* (Human)	92.3933
XP_055426971.1	*Bubalus carabanensis* (Swamp buffalo)	92.3933
XP_054347447.1	*Pongo pygmaeus* (Bornean orangutan)	92.3933
NP_001037841.1	*P.troglodytes* (Chimpanzee)	92.2078
XP_037699598.1	*Choloepus didactylus* (Linnaeus’s two-toed sloth)	92.2078
XP_036764538.2	*Manis pentadactyla* (Chinese pangolin)	92.1933
XP_055245879.1	*Gorilla gorilla gorilla* (Western gorilla)	92.0223
NP_001108439.1	*Macaca mulatta* (Rhesus monkey)	92.0223
XP_026304665.1	*Piliocolobus tephrosceles* (Ugandan red colobus)	92.0223
XP_006913709.1	*Pteropus alecto* (Black flying fox)	92.0223
WAK97440.1	*Macaca fascicularis* (Crab-eating macaque)	91.8367
KAI5939203.1	*Manis javanica* (Sunda pangolin)	91.8367
XP_039707041.1	*Pteropus giganteus* (Indian flying fox)	91.8367
XP_015976184.1	*Rousettus aegyptiacus* (Egyptian fruit bat)	91.8367
XP_033075496.1	*Trachypithecus francoisi* (François’ langur)	91.6512
XP_010366471.1	*Rhinopithecus roxellana* (Golden snub-nosed monkey)	91.6512
XP_012332513.1	*Aotus nancymaae* (Nancy Ma’s night monkey)	91.6512
JAB45812.1	*Callithrix jacchus* (Common marmoset)	91.6512
XP_058522307.1	*Ochotona princeps* (American pika)	91.4657
XP_006160378.1	*Tupaia chinensis* (Chinese tree shrew)	91.4657
XP_030875660.1	*Leptonychotes weddellii* (Weddell seal)	91.2801
XP_003944896.1	*Saimiri boliviensis boliviensis* (Bolivian squirrel monkey)	90.9091
AKU36829.1	*Bos taurus* (Cattle)	90.8257
XP_052504467.1	*Budorcas taxicolor* (Takin)	90.7579
XP_060259320.1	*Ovis aries* (Sheep)	90.573
XP_040106078.1	*Oryx dammah* (Scimitar oryx)	90.5556
XP_035967185.1	*Halichoerus grypus* (Gray seal)	90.538
XP_053456939.1	*Nycticebus coucang* (Slow loris)	90.3704
XP_046304483.1	*Marmota monax* (Groundhog)	90.1487
XP_015362942.1	*Marmota marmota marmota* (Alpine marmot)	90.1487
XP_026271099.1	*Urocitellus parryii* (Arctic ground squirrel)	90.1487

## Data Availability

Data sharing is not applicable to this article as no new data were created or analyzed in this study.
